# Memory Specificity Training for Depression and Posttraumatic Stress Disorder: A Promising Therapeutic Intervention

**DOI:** 10.3389/fpsyg.2018.00419

**Published:** 2018-04-03

**Authors:** Mina N. Erten, Adam D. Brown

**Affiliations:** ^1^Department of Psychology, Sarah Lawrence College, Bronxville, NY, United States; ^2^Department of Psychiatry, New York University School of Medicine, New York, NY, United States

**Keywords:** autobiographical memory, posttraumatic stress disorder (PTSD), major depressive disorder, psychotherapy, cognition

## Introduction

Major Depressive Disorder (MDD) and Posttraumatic Stress Disorder (PTSD) place great burdens on individuals, families, and society (e.g., Kessler, 2000; Greenberg et al., 2015). Although considerable advances have been made in the treatment of these disorders, many patients do not benefit from current therapeutic interventions (e.g., Turner et al., 2008; Cuijpers et al., 2009; Kar, 2011; Roque, 2015), resulting in a growing interest in the translation of findings from basic cognitive-neuroscience to the treatment of mental health issues such as MDD and PTSD (Cuthbert and Insel, 2013).

Cognitive-neuroscience research may help to advance the efficacy of current interventions in the characterization, identification, modification of maladaptive cognitive process underlying MDD and PTSD. One cognitive alteration in MDD and PTSD that may serve as a therapeutic target is Overgeneralized Autobiographical Memory (OGM).

Autobiographical memories have been characterized as a subset of long-term memories comprised of episodic and semantic details, that are viewed as highly central to one's self-narrative and life story (Rubin, 1998; Conway and Pleydell-Pearce, 2000). Cognitive models [e.g., Self-Memory-System (SMS), Conway and Pleydell-Pearce, 2000] propose that past knowledge along with one's current and future goals influence the accessibility and content of autobiographical memories. The SMS model emphasizes that the *working self* (current self-views and goals) inhibit autobiographical memories that are discrepant or may threaten current self-views. In relation to OGM, a phenomenon in which individuals diagnosed with these syndromes exhibit difficulty retrieving specific personal memories, clinical theorists posited the now influential CaRFAX model identifying potential mechanisms underlying the disruptions in autobiographical memory retrieval in depression and PTSD (Williams et al., 2007). The authors suggest that maladaptive cognitive processes often observed in depression and PTSD such as, *Capture and Rumination*, Functional Avoidance, and impairments in Executive Control independently or interdependently impair the successful recall of specific autobiographic memories.

Over the past decade, considerable findings have now shown a consistent relation between depression and PTSD with OGM (Moore and Zoellner, 2007; Williams et al., 2007; for reviews see: Lapidow and Brown, 2015). OGM is considered to be a critical mechanism underlying the pathogenesis of MDD and PTSD, as it has been associated with impairments in processes that may aid in coping and therapeutic outcomes, such as social problem solving, executive control, and future thinking (Moore and Zoellner, 2007; Williams et al., 2007; Sumner et al., 2011; Lapidow and Brown, 2015). For example, studies have found that impairments in problem solving in among depressed and PTSD patients have been predicted by greater overgeneralized autobiographical memories (e.g., Goddard et al., 1996; Sutherland and Bryant, 2008). Furthermore, OGM has been found to be a risk factor for the onset, course, and prognosis of MDD and PTSD (e.g., for full reviews on the link between the onset, maintenance, and treatment of depression, PTSD and OGM see Moore and Zoellner, 2007; Sumner et al., 2010; Lapidow and Brown, 2015).

The role of OGM in MDD and PTSD may be, in part, due to the functions of specific autobiographical memories in emotion regulation, decision-making, and social support (Bluck et al., 2005). For example, recalling similar specific events may aid in a person's ability to contextualize, (re-)appraise, self-regulate, and respond to novel stressors. In addition, individuals with impairments in retrieving specific autobiographical memories may have difficulty building and maintaining social support networks, as autobiographical memories are linked with social bonding (Bluck et al., 2005). Furthermore, deficits in autobiographical memory specificity appear to maintain maladaptive cognitive processes commonly observed in MDD and PTSD such as hopelessness, avoidance of negative memories, and rumination (for a summary see Raes et al., 2009). Building on the robust associations between OGM and these disorders, researchers are now showing that increasing autobiographical memory specificity may be a novel therapeutic target in the treatment of PTSD and MDD.

## The MEmory specificity training

The MEmory Specificity Training (MEST) (Raes et al., 2009) is a new treatment aimed at helping patients with MDD and PTSD to generate more specific autobiographical memories. Although the number of sessions varies by study, patients are first given an overview of the disorder being targeted, along with an explanation of OGM, followed by several sessions in which they are given cue-words and then are asked to practice recalling specific personal memories, prior to a final session in which they review key concepts. Additionally, patients are given homework between sessions in which they are asked to recall specific memories in response to positive, negative, and neutral cue words.

To date, two studies have been conducted with the MEST, the first targeting the depression symptoms of Iranian adolescents who had lost their fathers during the war in Afghanistan with a 5-session MEST training. Depression was assessed post-training and at 2-month follow-up (Nesthat-Doost et al., 2013). The authors found that, compared to a non-active control group, the two groups did not differ immediately at post-training. However, at the 2-month follow-up, a large effect was found, in which those patients who received the MEST reported significantly lower levels of depression. In a second study in which the MEST was employed to target symptoms of PTSD, Iranian war veterans showed a significant decrease in PTSD symptoms at post-training and at follow-up compared to the control group (Moradi et al., 2014), however the observed effect was smaller than those found by Nesthat-Doost et al. (2013). Nevertheless, these preliminary findings indicate that increasing autobiographical memory specificity may help to reduce MDD and PTSD.

## Life review therapy

A similar intervention aimed at reducing overgeneralization for patients of depression is the Life Review/Reminiscence Therapy (LRT; Haight and Webster, 1995). LRT is carried out within individual or group settings for approximately six sessions, much like the MEST; but differs structurally, as the therapist facilitates specificity increase through questions designed to prompt recall toward specific memories, rather than in response to cue-words (ex. “What is the most pleasant situation that you remember from your childhood?”). The premise of the training is that a failure to integrate specific life experiences into one's self-narrative will facilitate depression symptoms (Serrano et al., 2012).

The LRT was first tested with samples of older adult patients suffering from clinical depression and patients were randomized to either LRT or no treatment (Serrano et al., 2004). The authors found that LRT led to a significant reduction in symptoms of depression at follow-up and significantly lower levels of hopelessness, improved life satisfaction, and more specific autobiographical memories. In a subsequent study, Serrano et al. (2012) tested the LRT in older adults in which patients either received LRT or Treatment as Usual (TAU). In this case, differences were not observed between treatment groups. There may be several reasons for the lack of efficacy, perhaps in part due to age-related difficulties in recalling specific autobiographical memories (Luo and Craik, 2008).

## Concreteness training

A third intervention targeting OGM is Concreteness Training, the objective of which is to improve specificity for negative experiences through daily training exercises. Given the strong link between OGM and avoidance and rumination, Concreteness Training aims to teach patients how to focus on the objective aspects of the event. In Concreteness Training, a 6-week intervention, patients are prompted to use imagery in recalling sensory detail pertaining to the event, notice the sequence in which the event occurs, and finally specify the steps necessary to advance it (Watkins et al., 2009). In a study comparing Concreteness Training, relaxation training, and TAU, there was a reduction in OGM and symptoms of depression overgeneralization compared to the relaxation group and TAU (Watkins et al., 2011).

Although preliminary, taken together, these outcomes suggest that increasing autobiographical memory specificity may aid in treating MDD and PTSD. There are several issues with memory specificity training, however, that if addressed, may further enhance the efficacy of this approach. First, some individuals with PTSD and MDD may not benefit from autobiographical memory specificity trainings because they aim to avoid negative or traumatic memories. Second, the presence of OGM may make it difficult for individuals to recall specific memories. Third, it may be difficult for some patients, especially older adults with MDD or PTSD, to recall specific distant autobiographical memories. Additionally, it remains somewhat unclear the specific mechanisms associated with these therapeutic benefits. For example, Moradi et al. (2014) proposed that increasing autobiographical memory specificity may help PTSD patients to more effectively contextualize and re-script trauma narratives. Relatedly, Nesthat-Doost et al. (2013) theorized that specificity training may have led to a reduction in the mechanisms proposed in the CaRFAX model, but this hypothesis warrants further research, as these processes were not tested in their trial. Other studies, such as the Concreteness Training, proposed a number of potential therapeutic mechanisms such as a reduction in cognitive self-biases as well as increases in self-efficacy and pro-active coping (Watkins et al., 2011).

A new line of experimental research with healthy individuals is shedding light on the mechanisms underlying the benefits of autobiographical memory specificity and offers a novel approach to increasing specificity (Madore and Schacter, 2014; Madore et al., 2014; Jing et al., 2016).

## The episodic specificity induction

The Episodic Specificity Induction (Madore et al., 2014) was based on the premise that remembering and imagining both rely heavily on episodic memory (Schacter and Addis, 2007, 2009). In this memory specificity induction, participants are first asked to watch a short neutral film. Following the film, individuals are randomized to either a memory specificity induction or control condition. In the memory specificity induction, the experimenter uses a modified version of the Cognitive Interview (Fisher and Geiselman, 1992), a forensic protocol employed to increase reliability in eye-witness testimonies, to elicit specific episodic details about the film (e.g., “What color hair did the woman have?”). In contrast, participants in the control condition are asked general questions about their impressions of the film (e.g., “Did you like the video?”).

To date, three studies have been conducted on the impacts of the Episodic Specificity Induction. The first examined autobiographical memory and future thinking specificity in younger and older adults following the induction or control conditions (Madore et al., 2014). These findings showed that both younger and older adults generated more specific memories and future imaginings (as measured by internal details) following the specificity induction. Previous work has shown that tasks assessing social-problem solving [e.g., Means-End Problem Solving Task (MEPS), Platt and Spivack, 1975] are correlated with autobiographical memory specificity (Goddard et al., 1996; Sidley et al., 1997; Raes et al., 2005; Williams et al., 2007; Sutherland and Bryant, 2008; Maccallum and Bryant, 2010). Interestingly, Madore and Schacter (2014) found that younger and older adults generated more episodic details following the specificity induction. These findings support the link between social problem solving and memory specificity and begin to demonstrate how increasing specificity leads to changes in processes that may aid in recovery from PTSD and MDD.

A third study further demonstrated the role of episodic specificity in a wide range of clinically-relevant tasks (Jing et al., 2016). In this case, it was examined whether increasing memory specificity improved one's ability to reappraise anticipated future worrisome events. Consistent with the previous studies, increasing memory specificity with the Cognitive Interview subsequently led to more effective appraisals and a decrease in negative emotion about the negative future events. More recently, the specificity method was used to examine whether the induction could foster the generation of alternative future scenarios (Jing et al., 2017). Participants were asked to imagine a series of personal negative future events, followed by either the specificity or control induction, and were then asked to generate plausible alternatives to the events. After the specificity induction participants generated more possible alternatives and rated the future event as less negative than the control condition, and there was a negative correlation between the number of alternates generated and the perceived negativity of the event. Again, these findings suggest that cognitive processes related to well-being, such as reappraisal and problem solving, draw on episodic memory, and strategies that increase episodic memory improve one's ability to engage in these other episodically-supported tasks.

Although these studies were conducted entirely with non-clinical samples, the use of the CI paired with a neutral video maybe of relevance to clinical populations. Here, the researchers demonstrated increasing autobiographical memory and future thinking specificity without directly asking patients to practice these memories. In some cases, those with depression and PTSD may have difficulties generating specific memories because of OGM. Additionally, the use of the neutral video does not require participants to recall potentially negative or distressing personal memories. Although the recovery of these patients may ultimately necessitate the specific recollection of these memories, one might imagine that some patients could benefit from this specificity induction before they are ready to recall these memories (or given the role of specificity in emotion regulation, the induction may subsequently help them to cope with the recall of specific autobiographical memories). Furthermore, the use of a proximate stimulus (the video), compared to asking about an old memory, increases the likelihood that that participant will succeed in recalling the memory target. This may be useful for both clinical populations and older adults.

## Discussion and conclusion

As depression and anxiety continue to increase globally, there is an urgent need to develop novel interventions to reduce these symptoms. Although very preliminary, strategies that target and reduce OGM may be important in the treatment of MDD and PTSD. Among the strategies that have been deployed thus far with clinical populations, the MEST seems particularly promising and warrants further research. In addition, studies with non-clinical participants are shedding important light on the mechanisms underlying processes that may aid in therapy and would benefit with clinical populations.

## Author contributions

ME and AB contributed equally to all relevant tasks related to this manuscript.

### Conflict of interest statement

The authors declare that the research was conducted in the absence of any commercial or financial relationships that could be construed as a potential conflict of interest.
